# Academic Procrastination in University Students: Conscientiousness and Domain-Specific Executive Function Associations

**DOI:** 10.3390/bs16081368

**Published:** 2026-08-10

**Authors:** Stella Tsermentseli, Claire P. Monks

**Affiliations:** 1 Developmental & Educational Psychology Laboratory, Pedagogical Department of Primary Education, University of Thessaly, 382 21 Volos, Greece; 2 Institute for Lifecourse Development, University of Greenwich, London SE10 9LS, UK; c.p.monks@gre.ac.uk

**Keywords:** academic procrastination, conscientiousness, executive functions, cognitive flexibility, self-regulation, university students, academic performance

## Abstract

Academic procrastination is a common and persistent difficulty in higher education and is widely conceptualized as a self-regulatory failure. Although conscientiousness is a robust predictor of procrastination, less is known about whether specific executive function (EF) components contribute to procrastinatory behavior beyond personality traits. The present study examined relationships among domain-specific EF, conscientiousness, academic procrastination, and academic performance indicators in 117 university students. Participants completed performance-based measures of EF (inhibition, cognitive flexibility, and working memory), along with self-report measures of procrastination and conscientiousness. Academic performance indicators included cumulative grade point average (GPA) as an index of academic achievement, as well as behavioral indicators of task completion (i.e., late or missing coursework and incomplete assignments). Conscientiousness accounted for substantial variance in academic procrastination; however, EF showed a domain-specific association. Specifically, poorer cognitive flexibility was uniquely associated with higher levels of academic procrastination, whereas inhibition and working memory were not independently related once conscientiousness was considered. Academic procrastination was consistently associated with poorer academic performance indicators across both achievement and task-completion outcomes. Hierarchical analyses further indicated that academic performance reflected overlapping but non-identical associations with conscientiousness, selective EF components, and academic procrastination. These findings support a domain-specific approach to EF in the study of academic procrastination and underscore the value of moving beyond global accounts of EF in higher education contexts.

## 1. Introduction

Academic procrastination, defined as the voluntary delay of intended academic tasks despite awareness of potential negative consequences, remains a highly prevalent and persistent problem in higher education (Steel, 2007). Evidence indicates that a substantial proportion of university students regularly delay the initiation or completion of academic tasks, even when such delays are likely to undermine academic performance and well-being (Balkis & Duru, 2016; Gareau et al., 2019; Steel & Klingsieck, 2016). Academic procrastination has been consistently associated with poorer academic outcomes, including lower grade point average (GPA), increased rates of late or missing assignments, and a greater likelihood of incomplete coursework (Gareau et al., 2019; Kim & Seo, 2015; Park & Sperling, 2012). Beyond academic performance, procrastination has also been linked to elevated stress, reduced academic engagement, and maladaptive coping strategies, all of which may compromise learning quality and academic persistence over time (Balkis & Duru, 2016; Sirois et al., 2003). As higher education increasingly emphasizes independent learning and self-directed study, understanding why students procrastinate remains a central challenge for research concerned with academic success and self-regulated learning.

Academic procrastination is widely conceptualized as a failure of self-regulation, reflecting difficulties in initiating, sustaining, and completing goal-directed behavior despite the intention to do so (Senécal et al., 1995; Steel, 2007). Self-regulation broadly involves managing thoughts and impulses in ways that support goal attainment (Baumeister et al., 1994). From a self-regulatory perspective, high academic performance requires the ability to maintain task goals, resist competing temptations, and regulate effort over extended periods, particularly in learning environments characterized by high autonomy and delayed reinforcement. Procrastination emerges when students struggle to translate academic intentions into timely action, producing an intention–action gap that is especially pronounced in self-directed academic contexts (Steel & Klingsieck, 2016). Contemporary models further suggest that procrastination reflects a dynamic interplay between motivational and cognitive control processes that can undermine action even when academic goals are endorsed (Chowdhury & Pychyl, 2018; Dewitte & Schouwenburg, 2002; Klingsieck et al., 2013; Svartdal et al., 2020; Tice et al., 2001).

Within this framework, procrastination can be examined alongside other manifestations of self-regulatory functioning, including dispositional tendencies (e.g., personality traits) and cognitive control capacities (e.g., executive functions). Personality traits capture relatively stable patterns of motivation and behavior, whereas executive functions (EF) reflect the cognitive capacity to regulate behavior in real time under academic demands (Friedman & Miyake, 2017; Hoyle & Gallagher, 2015). Distinguishing between these components is critical, as students may be motivated to succeed and endorse adaptive academic goals yet still struggle with task initiation or completion due to limitations in cognitive control. Academic procrastination therefore provides a useful lens through which to examine the relative contributions of dispositional and cognitive processes to academic behavior.

Among personality traits, conscientiousness has consistently emerged as the strongest and most robust predictor of academic procrastination. Conscientiousness reflects individual differences in organization, persistence, self-discipline, and goal-directed behavior, all of which are directly relevant to academic performance in higher education contexts (Costa & McCrae, 1992; Watson, 2001). Meta-analytic and large-scale studies have demonstrated a strong negative association between conscientiousness and procrastination, indicating that students who score lower on conscientiousness are substantially more likely to delay academic tasks (Steel, 2007; Steel & Klingsieck, 2016). Facet-level analyses further suggest that components such as self-discipline, orderliness, and impulse control are particularly relevant to procrastinatory behavior (Lee et al., 2006; Watson, 2001). More recent studies have replicated and extended these findings across diverse cultural and educational contexts, confirming that conscientiousness is reliably associated with lower levels of academic procrastination and better academic performance (Balkis & Duru, 2016; Dominguez-Lara et al., 2019; Gareau et al., 2019). Conscientiousness has also been shown to predict academically relevant behaviors such as timely assignment submission, persistence in academic tasks, and higher GPA, underscoring its educational significance (Kim & Seo, 2015). These findings position conscientiousness as a theoretically proximal and empirically robust trait-level correlate of academic procrastination.

Despite its strong predictive value, conscientiousness alone does not fully account for variability in academic procrastination. Conscientiousness reflects typical behavioral tendencies and motivational orientation but does not directly capture the cognitive control processes required for moment-to-moment regulation of academic behavior, particularly under conditions of distraction or cognitive load (Hoyle & Gallagher, 2015). Consequently, students who report relatively high conscientiousness may still experience difficulties with task initiation, sustained attention, or resistance to competing temptations in demanding academic contexts. Recent work has therefore emphasized the importance of examining cognitive processes, such as EF, alongside personality traits to better understand why some students procrastinate despite endorsing adaptive motivational characteristics (Chowdhury & Pychyl, 2018; Klingsieck et al., 2013; Wieland et al., 2021).

EF refers to a set of interrelated but separable cognitive processes that support the regulation of thought and behavior in the service of goal-directed activity, particularly in novel, complex, or cognitively demanding situations. According to the unity–diversity framework, executive functions comprise separable but interrelated cognitive processes that contribute differentially to goal-directed behavior (Miyake et al., 2000; Friedman & Miyake, 2017). This perspective suggests that examining specific executive domains, rather than treating executive functioning as a unitary construct, may provide a more precise understanding of academic procrastination. In educational contexts, these EF components are critical for managing competing academic demands, maintaining task goals over time, resisting distraction, and flexibly adjusting strategies in response to feedback or difficulty. Accordingly, academic procrastination, characterized by repeated failures to initiate or sustain academic tasks despite intention, has been increasingly linked to EF (Gustavson et al., 2015; Rabin et al., 2011; Rebetez et al., 2016). However, from a unity–diversity perspective, it remains unclear whether all EF components contribute equally to academic procrastination once dispositional self-regulation, particularly conscientiousness, is considered or whether procrastination is selectively associated with specific EF domains.

Initial empirical links between EF and procrastination emerged primarily from studies using self-report or global indices of EF. Seminal work by Rabin et al. (2011) demonstrated that higher levels of procrastination were associated with broader self-reported EF difficulties, including problems with planning, task initiation, organization, and self-monitoring. Similarly, Howell and Watson (2007) found that higher levels of procrastination were associated with less adaptive learning strategies and poorer self-regulatory learning behaviors. While these findings established an important conceptual association between self-regulatory functioning and procrastination, their reliance on self-report measures limited conclusions regarding the specific EF domains involved. Conceptual overlap between procrastination measures and EF questionnaires may inflate associations and obscure domain-specific mechanisms (Rabin et al., 2011; Toplak et al., 2013).

Subsequent research has increasingly employed performance-based EF tasks to clarify domain-specific associations, although findings have been mixed. Working memory has been proposed as relevant to procrastination because of its role in maintaining task goals, deadlines, and subgoals during complex academic activities. Some studies have reported modest associations between poorer working memory performance and higher procrastination (e.g., Gustavson et al., 2015), suggesting that difficulties in goal maintenance may increase susceptibility to distraction. These findings have led to the suggestion that working memory may be more critical for task execution than for task initiation, rendering it less central to procrastination.

Inhibitory control has also been widely theorized as central to procrastination, given that procrastination often involves prioritizing short-term rewards over long-term academic goals (Dewitte & Schouwenburg, 2002; Tice et al., 2001). Empirical support for this account, however, has been inconsistent. While some studies using go/no-go or stop-signal tasks have reported weak associations between poorer inhibition and higher procrastination (Rebetez et al., 2016), these effects frequently diminish or disappear once conscientiousness or trait self-control is included in the model (Gustavson et al., 2014). This pattern suggests substantial shared variance between inhibitory control and dispositional self-discipline, raising questions about inhibition’s unique contribution to procrastinatory behavior beyond personality.

Cognitive flexibility, the ability to shift mental sets, update strategies in response to feedback, and disengage from ineffective approaches, has received comparatively less empirical attention in the procrastination literature, despite its clear theoretical relevance. Cognitive flexibility may be particularly important for academic task initiation and re-engagement, as students must often revise plans, tolerate uncertainty, and return to tasks following delay or difficulty (Klingsieck et al., 2013; Miyake et al., 2000). Emerging evidence suggests that reduced flexibility may be associated with rigid avoidance patterns and sustained task delay (Svartdal et al., 2020; Wieland et al., 2021). However, few studies have examined cognitive flexibility using performance-based measures or tested its incremental contribution beyond conscientiousness, leaving its role in academic procrastination underexplored.

Recent evidence further supports the relevance of EF to procrastination while also suggesting that associations may vary across EF domains and individuals. For example, Liu and Zhang (2025) compared active procrastinators, passive procrastinators, and non-procrastinators and found significant differences in inhibition and updating performance, with passive procrastinators showing weaknesses in inhibitory control. Notably, the pattern of findings differed across procrastination subtypes, suggesting that procrastination may be associated with heterogeneous EF characteristics rather than a uniform pattern of EF difficulties. Similarly, Wang et al. (2025) reported that higher levels of trait procrastination were associated with poorer self-reported EF among university students and that EF mediated the relationship between procrastination and sleep quality. Together, these findings provide further evidence that EF is implicated in procrastinatory behavior while highlighting the importance of examining specific EF domains and measurement approaches rather than relying exclusively on global indicators of EF.

Taken together, existing research supports the view that procrastination is related to EF, but the nature of this relationship appears domain-specific and remains theoretically unresolved. Evidence is strongest for broad EF difficulties assessed via self-report, whereas performance-based findings for inhibition and working memory are often weak or attenuated when dispositional traits such as conscientiousness are considered (Gustavson et al., 2015; Rebetez et al., 2016). Cognitive flexibility is theoretically well positioned to support task initiation, feedback-based adjustment, and re-engagement after delay, processes that are central not only to procrastination but also to effective academic task completion, yet remains comparatively understudied using performance-based EF measures and models that test associations beyond conscientiousness (Svartdal et al., 2020; Wieland et al., 2021). Importantly, much of the existing literature in this field has focused on cumulative academic achievement, typically indexed by GPA, with less attention to behaviorally meaningful academic performance indicators such as late or missing assignments and incomplete coursework. These task-completion outcomes may represent more proximal behavioral manifestations of self-regulatory difficulty and may be particularly sensitive to procrastination-related processes in higher education contexts (Gareau et al., 2019; Richardson et al., 2012).

**Figure 1:**
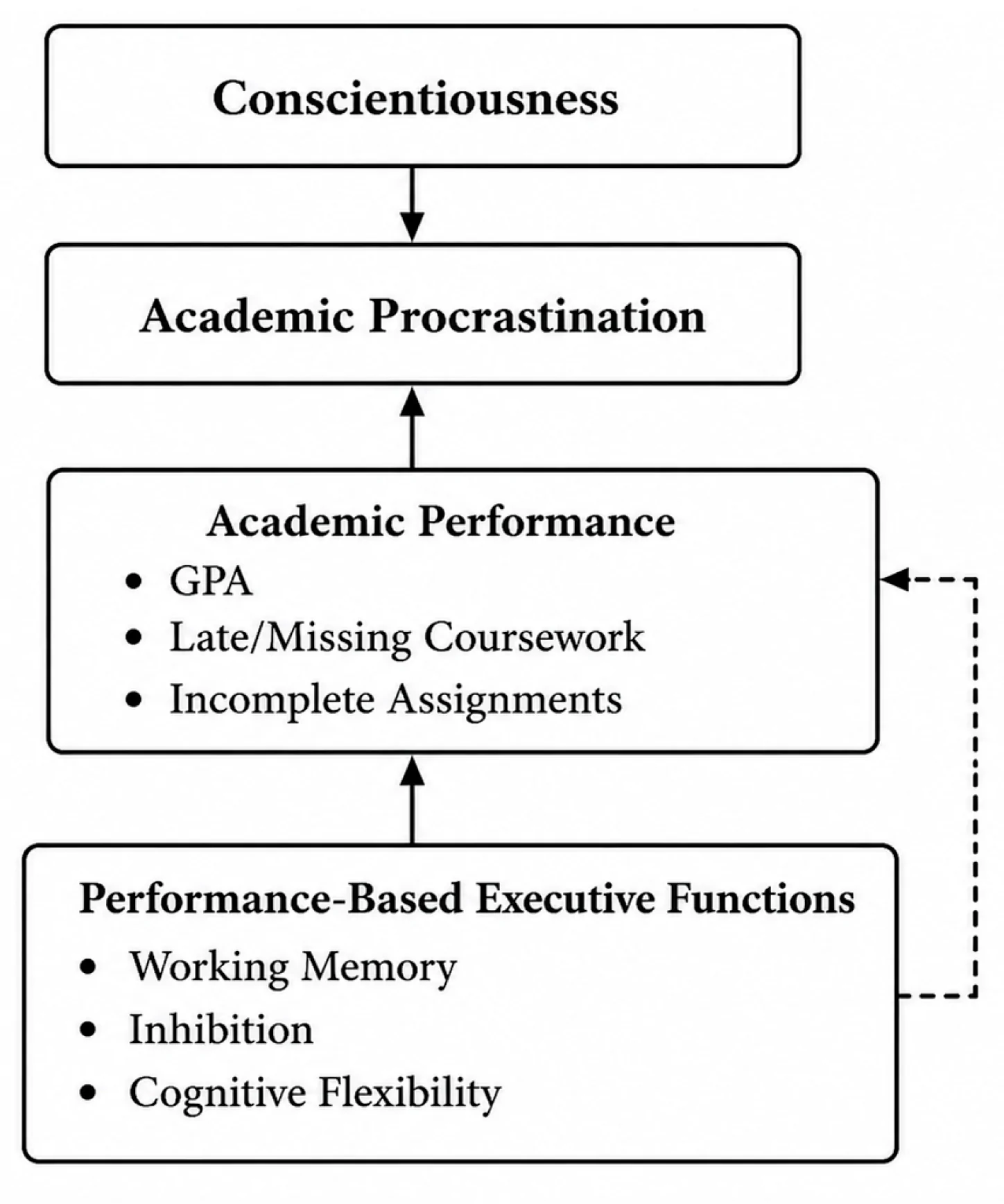
Conceptual framework of the present study. The figure illustrates the conceptual relationships examined among conscientiousness, performance-based executive functions (working memory, inhibition, and cognitive flexibility), academic procrastination, and academic performance indicators (GPA, late/missing coursework, and incomplete assignments). Executive functions were examined as predictors of both academic procrastination and academic performance, while accounting for conscientiousness.

Addressing these gaps, the present study examined working memory, inhibition, and cognitive flexibility as distinct EF domains in relation to procrastination among higher education students, while also accounting for conscientiousness as a key dispositional component of self-regulation. In contrast to much prior research that has relied on global or self-reported indices of EF, the present study adopted a domain-specific, performance-based approach to clarify whether specific EF components show unique associations with academic procrastination beyond conscientiousness. In addition, academic performance was operationalized using both cumulative academic achievement (i.e., GPA) and deadline-related academic outcomes as behavioral indicators of task completion (i.e., late or missing coursework and incomplete assignments), allowing examination of whether different academic outcomes reflect overlapping or distinct self-regulatory correlates. By jointly examining conscientiousness, domain-specific EF, and academic procrastination, the study aimed to (a) identify selective EF associations with procrastination, rather than assuming global EF involvement; (b) test the incremental contribution of EF domains beyond conscientiousness; and (c) clarify how academic procrastination and EF domains relate to multiple indicators of academic performance both at the level of direct associations and in terms of their incremental and relative contributions to cumulative academic achievement and behavioral indicators of task completion (see Figure 1. for a conceptual diagram on the study). This approach enables a more precise characterization of the self-regulatory processes underlying academic procrastination and its academic consequences.

## 2. Materials and Methods

### 2.1. Participants

One hundred and seventeen (*N* = 117) undergraduate students aged 18–30 years (M = 20.86, SD = 2.86) participated in the study. Participants were recruited from two universities in the United Kingdom (UK) using a convenience sampling approach. A convenience sampling approach was adopted because the study required individual laboratory-based assessment using performance-based EF tasks, making large-scale or probability sampling impractical within the available resources and recruitment period. The final sample consisted of 117 undergraduate students (70 females, 59.8%; 47 males, 40.2%). Inclusion criteria required participants to be between 18 and 30 years of age and fluent in English. Exclusion criteria included a self-reported history of special educational needs and disabilities (SEND) or current use of psychoactive medication because both may substantially influence performance on laboratory-based EF tasks and increase variability unrelated to the study aims. These criteria were applied to improve the internal validity of the study rather than to imply that these groups are inherently characterized by poorer EF. No participants were excluded following recruitment, either through self-withdrawal or investigator decision.

### 2.2. Measures

#### 2.2.1. Academic Procrastination (Trait Procrastination Expressed in Academic Functioning)

Academic procrastination was assessed using the Lay General Procrastination Scale (Lay, 1986), a widely used self-report measure of trait-level procrastination. The scale assesses individuals’ habitual tendency to delay the initiation or completion of tasks across everyday contexts. Although the Lay General Procrastination Scale is not course-specific, it was used in the present study to capture stable individual differences in procrastination that are expressed in academic functioning, rather than situational or task-specific fluctuations in academic delay. Trait procrastination, as indexed by this measure, has been shown to generalize to academic contexts and to robustly predict academic delay behaviors and academic performance outcomes in higher education samples (Steel, 2007; Kim & Seo, 2015; Steel & Klingsieck, 2016). Accordingly, in the present study, the scale was used to index individuals’ general tendency toward task delay as manifested in academic settings. The scale consists of 20 items (e.g., “I often find myself performing tasks that I had intended to do days before”), rated on a 5-point Likert scale, with higher scores indicating greater procrastination. Item scores were summed to produce a total score ranging from 20 to 100, with several items reverse-scored prior to computing total scores. The Lay General Procrastination Scale has demonstrated good internal consistency (Cronbach’s α = 0.82) and adequate test–retest reliability (r = 0.80; Ferrari, 1989). In the present sample, internal consistency was satisfactory (Cronbach’s α = 0.79).

#### 2.2.2. Conscientiousness

Conscientiousness was measured using a 20-item scale from the International Personality Item Pool (IPIP; Goldberg et al., 2006), assessing domain-level conscientiousness within the Five-Factor Model. Participants rated how accurately each statement described them on a 5-point Likert scale ranging from 1 (very inaccurate) to 5 (very accurate). Example items include “I am always prepared” and “I do just enough work to get by.” Negatively worded items were reverse-scored, and item responses were summed to yield a total conscientiousness score, with higher scores reflecting greater levels of conscientiousness. Internal consistency in the present sample was acceptable (Cronbach’s α = 0.75).

#### 2.2.3. Academic Performance Indicators

Academic functioning was assessed using multiple indicators to capture distinct aspects of academic performance. Grade point average (GPA) was included as an index of cumulative academic achievement, whereas late or missing coursework and incomplete assignments were included as more proximal behavioral indicators of task engagement and self-regulation during students’ current program of study. Participants reported their cumulative academic grade as a percentage (0–100%), consistent with UK grading conventions. Academic indicators were self-reported due to data protection constraints; an approach commonly used in higher education research. These values were converted to a 5-point GPA scale based on standard UK degree classification bands to facilitate analysis and comparability across outcomes. Grades were recoded as follows: 1 = Fail (<40%), 2 = Third Class (40–49%), 3 = Lower Second Class (50–59%), 4 = Upper Second Class (60–69%), and 5 = First Class (≥70%). This conversion preserved the ordered structure of academic achievement while providing a standardized GPA metric for analysis.

In addition to GPA, academic task-completion indicators were used to index behavioral engagement with coursework demands. Participants reported the number of late or missing coursework submissions and the number of assignments not completed by the specified deadlines since the start of their course. These indicators captured proximal academic behaviors related to task initiation, persistence, and completion, rather than overall achievement. Together, the inclusion of both GPA and task-completion indicators provided a comprehensive assessment of academic functioning, encompassing both performance outcomes and self-regulatory behaviors.

#### 2.2.4. Performance-Based Measures of Executive Function

Executive functions were assessed using three computerized tasks implemented in the Psychology Experiment Building Language (PEBL; Mueller & Piper, 2014). For each task, a single primary outcome measure was selected a priori based on established recommendations in the literature.

Inhibition was assessed using the Stop-Signal Task (SST; Logan et al., 1984), a well-established performance-based measure of response inhibition. Participants were instructed to respond as quickly and accurately as possible to left- and right-pointing arrows by pressing designated keys. On a subset of trials, the arrow stimulus was presented with a red circle, signaling that participants should withhold their motor response. Practice trials were administered until participants achieved 20 consecutive correct responses to ensure task comprehension before proceeding to the test phase. The test phase consisted of 50 trials. Inhibition was indexed by the number of failed stop trials, reflecting instances in which participants were unable to successfully inhibit their response following presentation of the stop signal. Higher scores indicated poorer inhibitory control. Because the present task did not implement the adaptive staircase procedure typically used to obtain reliable estimates of stop-signal reaction time (SSRT), inhibitory control was indexed by the number of failed stop trials. Although SSRT is generally considered the preferred outcome measure in stop-signal paradigms, failed stop trials provide a direct and readily interpretable behavioral index of inhibitory failures under the task parameters used in the present study. This approach also avoids the estimation assumptions required for SSRT calculation, which may be problematic when the methodological requirements for reliable SSRT estimation are not fully met (Verbruggen et al., 2019).

Cognitive flexibility was assessed using the Wisconsin Card Sorting Task (WCST; Berg, 1948), a widely used performance-based measure of set shifting and adaptive cognitive control, as implemented in the Psychology Experiment Building Language (PEBL; Mueller & Piper, 2014). Although perseverative errors are commonly interpreted as indicators of cognitive flexibility, performance on the WCST is also influenced by related executive processes, including feedback monitoring, rule maintenance, and strategic responding. Participants were required to match a target card to one of four reference cards based on color, number, or shape. The correct sorting rule had to be inferred through trial-and-error feedback. Once a sorting rule was acquired, it remained in effect for a period before changing without warning, requiring participants to monitor feedback and flexibly adjust their response strategy. The task consisted of 60 test trials, excluding practice trials. Cognitive flexibility was indexed by the number of perseverative errors, defined as continued responding according to a previously correct but no longer relevant sorting rule. Higher perseverative error scores indicated poorer cognitive flexibility.

Working memory was assessed using a Digit Span Backward task, administered via the Psychology Experiment Building Language (PEBL; Mueller & Piper, 2014). Participants were presented with sequences of digits displayed sequentially on the screen and were instructed to recall each sequence in reverse order. Sequence length increased incrementally across trials. At each span length, three trials were administered, and progression to the next span length required correct recall on at least two trials. The task was terminated following two consecutive failures at a given span length. The primary outcome measure was the maximum backward digit span successfully recalled, with lower scores indicating poorer working memory performance.

### 2.3. Procedure

Participants completed the study during an approximately 45 min one-to-one laboratory session conducted at the University of Greenwich, UK. The session began with the administration of the EF tasks, followed by the self-report questionnaires. EF tasks were administered in a fixed order to ensure procedural consistency across participants. Although this approach may have introduced order or fatigue effects, it reduced procedural variability between participants. Participants were offered an optional break prior to completing the self-report measures of academic procrastination and conscientiousness, along with demographic information and academic performance indicators. All participants provided written informed consent prior to participation, and ethical approval for the study was granted by the University of Greenwich Departmental Research Ethics Committee.

### 2.4. Data Analysis

Data were analysed using IBM SPSS Statistics (Version 26). Data were screened for accuracy, missing values, and outliers. No missing data were identified for the primary study variables. Descriptive statistics were computed for all EF measures, self-report questionnaires, and academic performance indicators. Distributional assumptions were evaluated using visual inspection of histograms, skewness and kurtosis indices, and Shapiro–Wilk tests. As the majority of variables approximated normal distributions, parametric analyses were deemed appropriate. Firstly, Pearson’s r correlations were conducted to examine bivariate associations among EF domains, academic procrastination, conscientiousness, and academic performance indicators. Subsequently, to assess whether EF explained variance in academic procrastination beyond conscientiousness, hierarchical multiple regression analyses were performed. Age and gender were entered as control variables in the first step, followed by conscientiousness in the second step and EF domains entered simultaneously in the final step to evaluate incremental variance explained. Associations between academic procrastination and academic performance indicators were examined using regression models appropriate to the distribution of each outcome. Linear regression was used to predict GPA. Although the GPA variable was derived from five ordered UK degree-classification categories, it comprised five ordered levels of academic achievement and showed no substantial departures from normality. It was therefore treated as an approximately continuous outcome for the hierarchical linear regression analyses. Because late or missing coursework and incomplete assignments constituted count data and exhibited overdispersion, negative binomial regression models were estimated for these outcomes. To examine the relative contributions of conscientiousness, EF, and academic procrastination to academic performance, hierarchical linear regression and nested negative binomial regression models were conducted, with model comparisons based on changes in explained variance and model fit. All statistical tests were two-tailed, and significance was set at *p* < 0.05.

## 3. Results

### 3.1. Descriptives and Correlational Analyses

Descriptive statistics for all EF measures, self-report variables, and academic performance indicators are presented in Table 1. Overall, participants demonstrated moderate levels of academic procrastination and conscientiousness, alongside relatively low mean frequencies of late or missing coursework and incomplete assignments.

Pearson correlation analyses were conducted to examine associations among EF measures, academic procrastination, conscientiousness, and academic performance indicators (see Table 2). As shown in Table 2, academic procrastination was associated with poorer academic functioning, including lower GPA, higher rates of late or missing coursework, and a greater number of incomplete assignments. Academic procrastination was also strongly negatively associated with conscientiousness. With respect to EF, poorer cognitive flexibility, indexed by a greater number of perseverative errors on the WCST, was associated with higher levels of academic procrastination, whereas inhibitory control showed a weaker association and working memory was not significantly related to procrastination. Cognitive flexibility was also associated with higher rates of late or missing coursework and incomplete assignments. Conscientiousness was consistently associated with more adaptive academic performance indicators, including higher GPA and fewer late or missing coursework and incomplete assignments.

### 3.2. Executive Functions Beyond Conscientiousness in Academic Procrastination

To examine whether EF domains explained variance in academic procrastination beyond conscientiousness, a hierarchical multiple regression analysis was conducted with procrastination as the dependent variable. Preliminary analyses indicated that assumptions of linearity, normality of residuals, homoscedasticity, and absence of problematic multicollinearity were adequately met. Tolerance values ranged from 0.43 to 0.86 and variance inflation factor (VIF) values ranged from 1.16 to 2.34, indicating no evidence of problematic multicollinearity among predictors. Age and gender were entered as control variables in block 1. Gender was dummy-coded for the regression analyses (0 = male, 1 = female). Conscientiousness was entered in block 2, and working memory, inhibition, and cognitive flexibility were entered simultaneously in block 3. The regression results are presented in Table 3.

After controlling for age and gender, conscientiousness accounted for a substantial proportion of variance in academic procrastination, explaining an additional 54.3% of the variance, ΔR^2^ = 0.543, F(1, 113) = 138.60, *p* < 0.001. Importantly, the inclusion of EF variables in block 3 explained a further 4.4% of variance in academic procrastination beyond conscientiousness and demographic controls, ΔR^2^ = 0.044, F(3, 110) = 4.03, *p* = 0.009. Although modest in magnitude, this increment indicates that domain-specific EF processes explained a small but statistically significant proportion of variance in academic procrastination beyond conscientiousness. In the final model, lower conscientiousness (β = −0.66, *p* < 0.001) and poorer cognitive flexibility, indexed by greater perseverative errors (β = 0.24, *p* < 0.001), uniquely predicted higher levels of academic procrastination. In contrast, inhibition and working memory did not emerge as significant unique predictors.

### 3.3. Academic Performance Indicators: Associations and Relative Contributions of Procrastination and Executive Functions

#### 3.3.1. Academic Performance Indicators: Associations Between Academic Procrastination and Academic Outcomes

To address Aim 3, separate regression models were estimated to examine associations between academic procrastination and multiple indicators of academic performance, including GPA, late or missing coursework, and incomplete assignments. A linear regression analysis indicated that academic procrastination was significantly associated with lower GPA after controlling for age and gender, B = −0.037, β = −0.59, *p* < 0.001 (see Table 4). Neither age nor gender were significantly associated with GPA. These results indicate that higher levels of academic procrastination are linked to poorer cumulative academic achievement.

Because late or missing coursework constituted count data and showed evidence of overdispersion, a negative binomial regression model was estimated. Academic procrastination was associated with a significantly higher rate of late or missing coursework submissions, IRR = 1.046, *p* < 0.001, indicating that each one-unit increase in procrastination score was associated with an approximately 4.6% increase in the expected rate of late or missing submissions. Age and gender were not significantly associated with this outcome. A second negative binomial regression model examined incomplete assignments. Academic procrastination again emerged as a significant predictor, IRR = 1.051, *p* < 0.001, corresponding to an approximate 5.1% increase in the expected rate of incomplete assignments per unit increase in procrastination. Age and gender were not significantly associated with incomplete assignments. Full model estimates are presented in Table 4.

#### 3.3.2. Academic Performance Indicators: Relative and Incremental Contributions of Procrastination and Executive Functions

Taken together, Section 3.3.1 analyses indicate that academic procrastination is robustly associated with multiple indicators of academic performance, including both GPA and deadline-related academic outcomes (late or missing coursework and incomplete assignments). However, these models do not clarify whether procrastination accounts for unique variance in academic outcomes beyond conscientiousness and domain-specific EF, nor whether EF contributes to academic performance independently of procrastination. Accordingly, hierarchical and nested modelling approaches were employed to examine the relative and incremental contributions of conscientiousness, EF domains, and academic procrastination to academic performance indicators, allowing differentiation between overlapping and distinct sources of variance in academic achievement and behavioral indicators of task completion.

A hierarchical linear regression was conducted to examine the relative contributions of conscientiousness, EF domains, and academic procrastination to GPA. Results are presented in Table 5. Because the primary aim of the analysis was to evaluate the incremental contribution of successive predictor blocks, the table summarizes model-comparison statistics rather than individual regression coefficients. At Block 1, conscientiousness, controlling for age and gender, accounted for a substantial proportion of variance in GPA (R^2^ = 0.363). The addition of EF domains at Block 2 explained a further 7.2% of variance beyond conscientiousness and demographic factors (ΔR^2^ = 0.072, ΔF = 4.70, *p* = 0.004). At Block 3, academic procrastination accounted for an additional 2.2% of unique variance beyond conscientiousness and EF domains (ΔR^2^ = 0.022, ΔF = 4.46, *p* = 0.037). The increase in explained variance at Block 2 reflects the combined contribution of EF domains entered simultaneously rather than unique associations of individual EF components with GPA. In the fully adjusted model, higher levels of academic procrastination were associated with lower GPA, indicating that procrastination explained variance in academic achievement beyond conscientiousness and the combined contribution of domain-specific EF measures.

For late or missing coursework, nested model comparisons indicated that adding EF domains and academic procrastination did not significantly improve model fit beyond conscientiousness (likelihood ratio tests, *ps* > 0.26), suggesting that conscientiousness accounted for most of the explainable variance in timely submission behavior. In contrast, for incomplete assignments, adding academic procrastination significantly improved model fit beyond conscientiousness and EF domains, Δχ^2^ (1) = 4.21, *p* = 0.040. Changes in the direction and magnitude of the procrastination coefficient in the fully adjusted model likely reflect shared variance with conscientiousness and should therefore be interpreted cautiously. Late or missing coursework and incomplete assignments constituted count outcomes with positively skewed distributions and evidence of overdispersion. Negative binomial regression models were therefore estimated. Full parameter estimates and model-fit statistics for all nested models are presented in Table 6.

For late or missing coursework, Model 1 indicated that higher conscientiousness was associated with a lower expected rate of late or missing submissions, IRR = 0.946, 95% CI [0.925, 0.968], *p* < 0.001. Female students also had a lower expected rate than male students in Model 1, IRR = 0.468, 95% CI [0.252, 0.868], *p* = 0.016, although this association was attenuated after the EF variables were included. Adding working memory, inhibition, and cognitive flexibility did not significantly improve model fit, Δχ^2^(3) = 3.97, *p* = 0.265. Academic procrastination provided no further improvement, Δχ^2^(1) = 0.01, *p* = 0.923. In the fully adjusted model, conscientiousness remained significantly associated with fewer late or missing submissions, whereas the EF measures and academic procrastination were not uniquely associated with this outcome.

For incomplete assignments, higher conscientiousness was associated with a lower expected rate in Model 1, IRR = 0.922, 95% CI [0.894, 0.951], *p* < 0.001. Adding the three EF domains did not significantly improve overall model fit, Δχ^2^(3) = 4.76, *p* = 0.190. The subsequent addition of academic procrastination significantly improved fit relative to Model 2, Δχ^2^(1) = 4.21, *p* = 0.040. In the fully adjusted model, conscientiousness remained negatively associated with incomplete assignments, IRR = 0.902, 95% CI [0.857, 0.949], *p* < 0.001, while greater WCST perseverative errors, indicating poorer cognitive flexibility, were associated with a higher expected rate of incomplete assignments, IRR = 1.095, 95% CI [1.020, 1.176], *p* = 0.013. The Wald test for the procrastination coefficient was marginal, IRR = 0.951, 95% CI [0.904, 1.001], *p* = 0.054, despite the significant likelihood-ratio comparison for its addition. This difference reflects the fact that the likelihood-ratio test evaluates improvement in overall model fit, whereas the Wald test evaluates the individual coefficient conditional on the other correlated predictors.

## 4. Discussion

The present study provides evidence that EF relate to academic procrastination in a selective, domain-specific manner beyond conscientiousness. Among EF, poorer cognitive flexibility, indexed by greater perseverative errors on the WCST, but not inhibition or working memory, was uniquely associated with higher academic procrastination once conscientiousness was taken into account. Academic procrastination, in turn, was consistently associated with poorer academic functioning, including lower GPA and greater difficulty meeting coursework requirements. Finally, academic performance reflected overlapping yet distinct contributions of conscientiousness and academic procrastination, with EF domains showing selective relevance for academic procrastination rather than direct associations with academic performance.

Consistent with a substantial body of prior research, conscientiousness emerged as the most robust correlate of academic procrastination. Conscientiousness, conceptualized as a stable dispositional tendency toward organization, persistence, reliability, and goal-directed behavior, has been repeatedly identified as the strongest personality correlate of procrastination across educational contexts (Steel & Klingsieck, 2016; Svartdal & Steel, 2017). These characteristics are particularly relevant in higher education environments that require students to independently manage time, sustain effort across extended periods, and initiate tasks in the absence of immediate external structure. Within contemporary self-regulation frameworks, conscientiousness is understood as a trait-level component of self-regulation, shaping individuals’ typical behavioral patterns across situations rather than their moment-to-moment cognitive control capacity (Friedman & Miyake, 2017; Hoyle & Gallagher, 2015). Students higher in conscientiousness tend to engage in proactive planning, adhere more consistently to deadlines, and persist when tasks are effortful, whereas lower conscientiousness is associated with greater susceptibility to distraction, inconsistent engagement, and avoidance of aversive tasks (Kim & Seo, 2015; Steel & Klingsieck, 2016). Importantly, conscientiousness captures motivational orientation and behavioral consistency rather than the cognitive mechanisms that support real-time behavioral adjustment. The strong association between conscientiousness and academic procrastination observed in the present study supports the view that procrastination is closely associated with enduring dispositional tendencies toward self-discipline and task commitment, rather than reflecting only situational pressures or transient failures of control (Svartdal et al., 2020). The magnitude of this association further supports the view that procrastination represents a central behavioral manifestation of dispositional self-regulation.

At the same time, conscientiousness did not fully account for individual differences in academic procrastination, consistent with theoretical accounts suggesting that motivational dispositions alone are insufficient to explain persistent failures to translate intentions into action, particularly under conditions of uncertainty, stress, or task aversion (Chowdhury & Pychyl, 2018; Klingsieck et al., 2013). Consistent with this view, the present study demonstrated that EF relates to academic procrastination in a selective and domain-specific manner. This finding extends recent evidence linking trait procrastination to broader EF difficulties in university students (Wang et al., 2025) by suggesting that the association may be driven more strongly by specific executive domains than by EF globally. When multiple EF domains were examined simultaneously alongside conscientiousness, only poorer cognitive flexibility, indexed by a greater number of perseverative errors on the WCST, showed a unique association with academic procrastination. Perseverative errors reflect difficulty abandoning previously reinforced but no longer effective response strategies and adapting behavior in response to changing feedback (Miyake et al., 2000; Friedman & Miyake, 2017). Accordingly, students who experience greater difficulty updating their behavior and shifting away from ineffective response patterns may also be more likely to exhibit persistent academic procrastination.

From this perspective, cognitive flexibility may be particularly relevant to procrastination because re-engaging with an academic task following an initial delay requires behavioral updating. Students must recognise that continued avoidance is no longer adaptive, disengage from the current response pattern, revise their behavioral strategy, and redirect their behaviour toward the intended academic goal. Greater perseverative responding may make this transition more difficult, allowing an initial episode of delay to develop into a more persistent pattern of task avoidance. This interpretation is consistent with EF dysfunction accounts that emphasize impaired behavioral updating and difficulty shifting away from maladaptive response patterns (Klingsieck et al., 2013; Sirois & Pychyl, 2016).

These findings should also be interpreted within the broader distinction between laboratory-based and everyday manifestations of EF. Although the WCST assesses the ability to infer changing rules, monitor feedback, and shift response strategies under structured laboratory conditions, behavioral flexibility in everyday life is additionally shaped by motivational, emotional, personality, and contextual influences (Toplak et al., 2013; Friedman & Miyake, 2017). Accordingly, performance on the WCST should be viewed as one indicator of executive control rather than as a comprehensive measure of an individual’s behavioral adaptability.

The absence of unique associations for inhibition and working memory is also informative. Although both processes are important for successful task execution, they may be less central to the initiation and re-initiation difficulties that characterize academic procrastination. Consistent with this interpretation, previous studies have shown that associations between inhibition, working memory, and procrastination are substantially reduced once conscientiousness or trait self-control is taken into account (Gustavson et al., 2015; Rebetez et al., 2016). Together, these findings suggest considerable conceptual and functional overlap between dispositional self-discipline and certain effortful control processes while highlighting cognitive flexibility as a more distinct cognitive correlate of procrastinatory behavior.

More broadly, the present findings help refine EF accounts of academic procrastination by identifying poorer cognitive flexibility as a specific domain of relevance. Much of the earlier literature linking EF to procrastination relied on self-report measures or global indices of EF, which may inflate associations through shared method variance and obscure domain-specific effects (Rabin et al., 2011; Toplak et al., 2013). By employing performance-based measures and examining EF domains simultaneously, the present study provides clearer evidence that procrastination is not uniformly associated with EF. Instead, difficulties in shifting appear particularly relevant, aligning with theoretical accounts that conceptualize procrastination as a difficulty in flexibly regulating behavior over time, especially in the context of negative affect, uncertainty, or perceived task difficulty (Sirois et al., 2003; Klingsieck et al., 2013). From this perspective, cognitive flexibility may be especially important at moments when students must overcome initial resistance or re-engage with tasks following avoidance, contributing to the persistence of procrastinatory behavior.

Beyond its dispositional and cognitive correlates, academic procrastination was consistently associated with poorer academic performance across multiple indicators. Procrastination was related not only to lower overall academic achievement (as measured by GPA) but also to behavioral indicators of academic engagement, including late or missing coursework and incomplete assignments. Examining these outcomes together provides a more comprehensive understanding of how procrastination undermines academic success in higher education contexts (Gareau et al., 2019; Richardson et al., 2012). Although GPA is a widely used indicator of academic achievement, it reflects performance accumulated across multiple semesters and is therefore influenced by numerous educational and contextual factors beyond current executive functioning and procrastination. In contrast, late or missing coursework and incomplete assignments provide more proximal behavioral indicators of students’ current self-regulatory functioning. The association between procrastination and lower achievement is well documented and underscores the long-term academic consequences of repeated task delay (Kim & Seo, 2015). Nevertheless, achievement outcomes alone may not fully capture the behavioral pathways through which procrastination influences academic functioning. Behavioral indicators such as missed deadlines and incomplete coursework, which capture more proximal manifestations of self-regulatory difficulty, are particularly relevant in autonomous learning environments. Late or missing coursework reflects difficulties with timely task initiation and completion, whereas incomplete assignments indicate more sustained disengagement from academic requirements. These behaviors are strongly associated with increased academic stress, delayed progression, and heightened risk of attrition (Richardson et al., 2012). The present findings therefore extend prior work by demonstrating that procrastination is linked not only to reduced academic achievement but also to failures in task engagement and completion. Nevertheless, because the present study was cross-sectional, these associations should not be interpreted as reflecting a unidirectional causal pathway. It is equally plausible that poorer academic performance contributes to subsequent task avoidance through processes such as reduced academic self-efficacy or increased academic anxiety, reinforcing procrastinatory behavior over time.

The final aim of the study was to examine how conscientiousness, EF domains, and academic procrastination jointly relate to academic performance. Although cognitive flexibility showed selective relevance for academic procrastination, EF domains were examined jointly in performance models to assess whether broader cognitive control capacity contributed to academic outcomes beyond dispositional traits and procrastination. The findings indicate that different academic outcomes are shaped by distinct configurations of dispositional traits, cognitive control processes, and behavioral tendencies. Overall academic achievement reflected the combined contributions of conscientiousness, the shared variance captured by EF domains, and procrastination. In contrast, deadline adherence was more closely aligned with conscientiousness, suggesting that timely submission behavior may be primarily associated with trait-level tendencies toward organization and persistence (Steel & Klingsieck, 2016; Richardson et al., 2012). Incomplete assignments showed a different pattern. Although academic procrastination improved overall model fit, its individual association was only marginal after adjustment for conscientiousness and EF. Poorer cognitive flexibility remained independently associated with incomplete assignments. Given the shared variance among these constructs, these findings should be interpreted cautiously and require replication. These findings suggest that academic procrastination does not fully account for the contribution of EF to academic task completion. Rather, conscientiousness, cognitive flexibility, and procrastination appear to represent overlapping but distinct aspects of self-regulation. Conscientiousness reflects relatively stable dispositional tendencies toward organization and persistence, whereas cognitive flexibility may support the implementation of goal-directed behavior by enabling students to adapt strategies, disengage from ineffective responses, and re-engage with academic tasks following setbacks or changing demands. Academic procrastination may therefore coexist with these executive processes rather than fully explain their contribution, reflecting the behavioral manifestation of broader self-regulatory functioning. Together, these findings highlight the importance of distinguishing among academic outcomes rather than treating academic performance as a unitary construct. Achievement, deadline adherence, and task completion represent related but distinct facets of academic functioning, each shaped by partially overlapping self-regulatory mechanisms (Svartdal et al., 2020).

Taken together, the present study contributes to the academic procrastination literature in three ways. First, it provides evidence that EF are associated with procrastination in a selective rather than uniform manner, with cognitive flexibility emerging as the only EF domain that showed a unique association with procrastination beyond conscientiousness. This finding extends previous work suggesting that different EF domains may show differential relationships with procrastination (Gustavson et al., 2015; Rebetez et al., 2016). Second, by employing performance-based measures of EF and testing their incremental contribution beyond a well-established personality correlate, the study offers a more stringent examination of cognitive mechanisms underlying procrastination than studies relying primarily on self-report measures of EF (Rabin et al., 2011; Toplak et al., 2013). Third, by examining both GPA and behavioral indicators of task completion, the findings demonstrate that different academic outcomes reflect overlapping but partially distinct self-regulatory processes, extending previous research that has focused predominantly on GPA as an indicator of academic performance (Gareau et al., 2019; Richardson et al., 2012). Collectively, these findings refine contemporary self-regulation accounts of procrastination by integrating domain-specific EF processes with dispositional models of self-regulation (Friedman & Miyake, 2017; Klingsieck et al., 2013).

From an applied perspective, the present findings suggest that approaches to addressing academic procrastination in higher education may benefit from extending beyond strategies focused primarily on motivation, time management, or self-discipline. Although strengthening conscientious behaviors remains important, the results indicate that these approaches may not fully address difficulties related to behavioral adjustment once task delay has occurred. The selective association between poorer cognitive flexibility and academic procrastination highlights the potential relevance of students’ capacity to disengage from avoidance-oriented responses and to re-engage with academic tasks following setbacks or initial delay. In autonomous learning environments, students are frequently required to revise plans, tolerate uncertainty, and adapt strategies over time; reduced flexibility in these contexts may contribute to the persistence of procrastinatory behavior even when academic goals are clearly endorsed. This interpretation is also consistent with emerging intervention research suggesting that educational programs targeting procrastination and EF can reduce procrastinatory behavior and improve EF abilities among university students (Cherrier et al., 2023).

Beyond intervention content, the findings also have implications for how academic procrastination is conceptualized and addressed within higher education support systems. Procrastination may be most usefully understood as a difficulty in behavioral regulation over time rather than solely as a motivational deficit, suggesting the value of assessment and support practices that focus on processes of disengagement and re-engagement. In addition, the presence of substantial variability in procrastination beyond conscientiousness indicates that students who appear motivated and organized may nevertheless experience significant difficulties with shifting, underscoring the importance of support approaches that extend beyond trait-based assumptions.

The present findings suggest that cognitive flexibility may represent a promising target for future intervention research. If supported by longitudinal and experimental studies, flexibility-oriented components could complement existing academic support practices. Such approaches emphasize adaptive strategy shifting, monitoring of task engagement, and effective use of feedback, rather than focusing exclusively on effort regulation or time allocation. Recent work in educational psychology has highlighted the value of metacognitive and self-regulated learning frameworks that support adaptive behavioral adjustment and re-engagement in response to academic difficulty or negative affect (Klingsieck et al., 2013; Panadero, 2017). From this perspective, supporting students’ ability to respond flexibly to academic challenges may help prevent initial delay from escalating into persistent task avoidance.

Importantly, the present findings do not suggest that EF broadly should be treated as a primary intervention target. Rather, they underscore the value of a domain-specific approach that considers how particular cognitive processes support distinct phases of academic behavior. Integrating flexibility-oriented elements into existing self-regulated learning or study-support initiatives may therefore offer a targeted and conceptually coherent approach to addressing academic procrastination in higher education settings.

### Limitations and Future Directions

Several limitations should be acknowledged. First, the cross-sectional design precludes conclusions regarding the directionality of the observed associations. Although the present findings are discussed within theoretical models in which EF and conscientiousness contribute to academic procrastination and, in turn, to academic performance, alternative or reciprocal pathways are equally plausible. For example, poorer academic performance may reduce academic self-efficacy, increase academic anxiety, or promote task avoidance, thereby reinforcing procrastinatory behavior over time. Such reciprocal processes are consistent with contemporary conceptualizations of procrastination as a dynamic self-regulatory process rather than a unidirectional causal sequence. Longitudinal and cross-lagged designs are needed to clarify the temporal ordering and potential feedback mechanisms linking EF, conscientiousness, procrastination, and academic performance. Second, the sample size was modest and may have limited statistical power to detect smaller associations, particularly for EF domains that showed weak or non-significant effects. Although the sample size was adequate for the regression models estimated in the present study, the substantial variance explained by conscientiousness may have reduced statistical power to detect very small incremental effects of individual EF domains. Consequently, the absence of significant associations for inhibition and working memory should be interpreted cautiously. Replication with larger samples would help determine whether these domains make small but meaningful contributions to academic procrastination beyond conscientiousness. Third, academic performance indicators were assessed using self-report measures, which may be subject to recall and reporting bias. Although self-reported academic measures are commonly used in higher education research and generally demonstrate acceptable correspondence with official records, reporting bias cannot be excluded. Future research should replicate these findings using institutional administrative data, such as registrar-verified GPA and learning management system submission records, to strengthen the ecological and external validity of the present findings. Fourth, the present study focused exclusively on performance-based measures of “cool” EF. Given growing evidence that “hot” EF, including affective and motivational decision-making processes, may play an important role in academic performance, future studies should integrate emotionally and motivationally salient tasks to more fully capture the self-regulatory demands of academic contexts (Sirois & Pychyl, 2016; Zelazo & Carlson, 2012). 

A further limitation concerns the assessment of executive functions. Each EF domain was represented by a single performance-based task, and cognitive flexibility was indexed by perseverative errors on the Wisconsin Card Sorting Task (WCST). Although WCST perseverative errors are commonly interpreted as reflecting cognitive flexibility, performance is influenced by multiple executive processes, including working memory, feedback monitoring, rule maintenance, and strategic problem solving. Consequently, the present findings should be interpreted as reflecting performance on a complex laboratory-based EF task rather than a process-pure or ecologically comprehensive measure of cognitive flexibility. Relatedly, EF and academic procrastination were assessed using different measurement modalities. While EF was measured using laboratory-based performance tasks that capture cognitive processes under controlled conditions, academic procrastination was assessed using a trait-like self-report questionnaire reflecting behavior across extended periods and contexts. These approaches assess related but distinct constructs and typically show only modest convergence (Toplak et al., 2013). Consequently, method variance may have attenuated associations between EF and academic procrastination, particularly for inhibition and working memory. Future research should combine multiple performance-based and self-report measures of EF, ideally within latent-variable models, to provide a more comprehensive understanding of the executive processes most closely associated with academic procrastination. Moreover, although age and gender were statistically controlled in all regression models, general cognitive ability (IQ) was not assessed. Because intellectual ability may contribute to both EF and academic achievement, future studies should examine whether the observed associations remain after accounting for standardized measures of IQ. Lastly, replication across diverse higher education contexts and student populations is needed to strengthen generalizability and further refine theoretical models of academic procrastination.

## 5. Conclusions

The present study offers a novel, theory-relevant contribution to the academic procrastination literature by testing a domain-specific EF account of procrastination within a self-regulation framework that has largely emphasized dispositional traits. Moving beyond global or self-reported EF indices that can obscure mechanisms through construct overlap, the study used performance-based measures of inhibition, working memory, and cognitive flexibility and evaluated their incremental associations with academic procrastination beyond conscientiousness, the most robust trait correlate of procrastination. The findings contribute to contemporary models by demonstrating a selective pattern: only cognitive flexibility was uniquely associated with academic procrastination once conscientiousness was considered, supporting the view that procrastinatory delay is linked less to generalized EF weakness and more to difficulty flexibly disengaging from avoidance and reorienting behavior toward task goals. By also linking procrastination to both cumulative achievement (GPA) and deadline-related task-completion indicators, the study extends behavioral self-regulation accounts to outcomes that more directly capture everyday academic engagement. Together, these results advance a layered conceptualization of self-regulation in higher education in which conscientiousness, domain-specific EF, and academic procrastination represent distinct yet related components, sharpening theoretical precision about which EF processes are most relevant for understanding academic task delay and its academic consequences.

## Figures and Tables

**Table 1:** Descriptive Statistics for Study Variables.

Variable	M	SD	Min–Max
Inhibition (failed stops)	21.19	10.25	7–60
Cognitive flexibility (perseverative errors)	11.71	6.79	5–35
Working memory (backward digit span)	8.18	2.68	0–15
Academic procrastination	61.79	14.58	34–91
Conscientiousness	67.56	13.03	30–94
Grade point average (GPA)	3.21	0.68	1–5
Late/missing coursework	0.94	1.60	0–8
Incomplete assignments	0.53	1.26	0–8

Note: M = mean; SD = standard deviation. Higher scores on inhibitory control (failed stops) and cognitive flexibility (perseverative errors) indicate poorer EF. Higher scores on academic procrastination indicate greater tendencies toward procrastination.

**Table 2:** Correlations among executive functions, procrastination, conscientiousness, and academic performance indicators.

Variable	1	2	3	4	5	6	7	8
Inhibition	—							
Cognitive flexibility	0.28 **	—						
Working memory	−0.19 *	−0.17	—					
Academic procrastination	0.23 *	0.45 ***	−0.12	—				
Conscientiousness	−0.18	−0.37 ***	0.14	−0.74 ***	—			
Grade point average	−0.21 *	−0.31 ***	0.16	−0.59 ***	0.51 ***	—		
Late/missing coursework	0.20 *	0.34 ***	−0.09	0.40 ***	−0.48 ***	−0.44 ***	—	
Incomplete assignments	0.14	0.29 **	−0.05	0.31 ***	−0.41 ***	−0.38 ***	0.61 ***	—

Note: * *p* < 0.05; ** *p* < 0.01; *** *p* < 0.001.

**Table 3:** Hierarchical multiple regression predicting academic procrastination.

Block	Predictor	B	SE B	β	t	*p*	R^2^	ΔR^2^
1	Age	−0.35	0.32	−0.07	−1.12	0.264	0.014	—
	Gender	3.17	1.84	0.22	1.72	0.088		
2	Conscientiousness	−0.74	0.08	−0.66	−9.79	<0.001	0.557	0.543 ***
3	Working memory	0.15	0.34	0.03	0.45	0.657	0.601	0.044 **
	Inhibition	−0.02	0.09	−0.02	−0.24	0.809		
	Cognitive flexibility	0.51	0.15	0.24	3.47	<0.001		

Note: B = unstandardized coefficient; SE B = standard error; β = standardized coefficient. ** *p* < 0.01; *** *p* < 0.001.

**Table 4:** Regression models examining associations between academic procrastination and academic performance indicators.

Outcome	Predictor	Estimate	95% CI	*p*
Grade point average	Academic procrastination	B = −0.037	[−0.047, −0.027]	<0.001
	Age	B = 0.01	[−0.01, 0.03]	0.311
	Gender	B = 0.06	[−0.06, 0.18]	0.314
Late/missing coursework	Academic procrastination	IRR = 1.046	[1.023, 1.070]	<0.001
	Age	IRR = 0.98	[0.94, 1.03]	0.482
	Gender	IRR = 0.88	[0.61, 1.27]	0.501
Incomplete assignments	Academic procrastination	IRR = 1.051	[1.021, 1.081]	<0.001
	Age	IRR = 0.97	[0.92, 1.03]	0.341
	Gender	IRR = 0.91	[0.59, 1.40]	0.669

Note: B = unstandardized regression coefficient; IRR = incidence rate ratio. Linear regression was used for GPA; negative binomial regression was used for late/missing coursework and incomplete assignments.

**Table 5:** Hierarchical regression predicting grade point average (GPA).

Block	Predictors	R^2^	ΔR^2^	F (df)	Δ*F*	*p*
1	Age, gender, conscientiousness	0.363	0.363	21.46 (3, 113)	—	<0.001
2	+Executive function domains (working memory, inhibition, cognitive flexibility)	0.436	0.072	14.17 (6, 110)	4.70	0.004
3	+Academic procrastination	0.458	0.022	13.16 (7, 109)	4.46	0.037

Note: *N* = 117. Executive function domains (working memory, inhibition, and cognitive flexibility) were entered simultaneously in Model 2. ΔR^2^ represents the additional variance explained by each successive model.

**Table 6:** Nested negative binomial regression models predicting late or missing coursework and incomplete assignments.

Outcome	Predictor	Model 1 IRR [95% CI]	*p*	Model 2 IRR [95% CI]	*p*	Model 3 IRR [95% CI]	*p*
Late or missing coursework	Age	0.981 [0.875, 1.100]	0.746	0.981 [0.875, 1.100]	0.745	0.981 [0.875, 1.100]	0.741
	Gender: female	0.468 [0.252, 0.868]	0.016	0.525 [0.276, 1.000]	0.050	0.525 [0.275, 1.001]	0.050
	Conscientiousness	0.946 [0.925, 0.968]	<0.001	0.955 [0.932, 0.979]	<0.001	0.954 [0.919, 0.990]	0.012
	Working memory	—	—	0.985 [0.872, 1.114]	0.814	0.985 [0.871, 1.114]	0.812
	Inhibition	—	—	1.013 [0.984, 1.044]	0.381	1.013 [0.983, 1.044]	0.386
	Cognitive flexibility	—	—	1.037 [0.992, 1.084]	0.106	1.038 [0.989, 1.090]	0.129
	Academic procrastination	—	—	—	—	0.998 [0.962, 1.035]	0.923
Incomplete assignments	Age	0.888 [0.750, 1.050]	0.164	0.868 [0.723, 1.042]	0.129	0.840 [0.691, 1.021]	0.080
	Gender: female	0.527 [0.249, 1.115]	0.094	0.619 [0.280, 1.370]	0.237	0.715 [0.313, 1.631]	0.425
	Conscientiousness	0.922 [0.894, 0.951]	<0.001	0.936 [0.906, 0.967]	<0.001	0.902 [0.857, 0.949]	<0.001
	Working memory	—	—	1.026 [0.868, 1.212]	0.764	1.034 [0.870, 1.228]	0.707
	Inhibition	—	—	1.017 [0.987, 1.049]	0.272	1.016 [0.984, 1.049]	0.320
	Cognitive flexibility	—	—	1.054 [0.996, 1.116]	0.070	1.095 [1.020, 1.176]	0.013
	Academic procrastination	—	—	—	—	0.951 [0.904, 1.001]	0.054

Note: IRR = incidence rate ratio; CI = confidence interval.

## Data Availability

The data presented in this study are not publicly available due to ethical restrictions related to participant consent and data protection. Data are available from the author upon reasonable request.

## Abbreviations

The following abbreviations are used in this manuscript:

EF	Executive Function
GPA	Grade Point Average
UK	United Kingdom
SEND	Special Educational Needs and Disabilities

## References

[B1-behavsci-16-01368] Balkis M., Duru E. (2016). Procrastination, self-regulation failure, academic life satisfaction, and affective well-being: Underregulation or misregulation form. European Journal of Psychology of Education.

[B2-behavsci-16-01368] Baumeister R. F., Heatherton T. F., Tice D. M. (1994). Losing control: How and why people fail at self-regulation.

[B3-behavsci-16-01368] Berg E. A. (1948). A simple objective technique for measuring flexibility in thinking. The Journal of General Psychology.

[B4-behavsci-16-01368] Cherrier S., Wattelez G., Ferrière S., Borst G. (2023). NeuroStratE: An educational neuroscience intervention to reduce procrastination behavior and improve executive planning function in higher students. Frontiers in Education.

[B5-behavsci-16-01368] Chowdhury S. F., Pychyl T. A. (2018). A critique of the construct validity of active procrastination. Personality and Individual Differences.

[B6-behavsci-16-01368] Costa P. T., McCrae R. R. (1992). Revised NEO Personality Inventory (NEO PI-R) and NEO Five-Factor Inventory (NEO-FFI): Professional manual.

[B7-behavsci-16-01368] Dewitte S., Schouwenburg H. C. (2002). Procrastination, temptations, and incentives: The struggle between the present and the future in procrastinators and the punctual. European Journal of Personality.

[B8-behavsci-16-01368] Dominguez-Lara S., Prada-Chapoñan R., Moreta-Herrera R. (2019). Gender differences in the influence of personality on academic procrastination in Peruvian college students. Acta Colombiana de Psicología.

[B9-behavsci-16-01368] Ferrari J. R. (1989). Reliability of academic and dispositional measures of procrastination. Psychological Reports.

[B10-behavsci-16-01368] Friedman N. P., Miyake A. (2017). Unity and diversity of executive functions: Individual differences as a window on cognitive structure. Cortex.

[B11-behavsci-16-01368] Gareau A., Chamandy M., Kljajic K., Gaudreau P. (2019). The detrimental effect of academic procrastination on subsequent grades: The mediating role of coping over and above past achievement and working memory capacity. Anxiety, Stress & Coping.

[B12-behavsci-16-01368] Goldberg L. R., Johnson J. A., Eber H. W., Hogan R., Ashton M. C., Cloninger C. R., Gough H. G. (2006). The International Personality Item Pool and the future of public-domain personality measures. Journal of Research in Personality.

[B13-behavsci-16-01368] Gustavson D. E., Du Y., Foerde K., Hewitt J. K., Friedman N. P. (2014). Genetic relations among procrastination, impulsivity, and goal-management ability: Implications for the evolutionary origin of procrastination. Psychological Science.

[B14-behavsci-16-01368] Gustavson D. E., Miyake A., Hewitt J. K., Friedman N. P. (2015). Understanding the cognitive and genetic underpinnings of procrastination: Evidence for shared genetic influences with goal management and executive function abilities. Journal of Experimental Psychology: General.

[B15-behavsci-16-01368] Howell A. J., Watson D. C. (2007). Procrastination: Associations with achievement goal orientation and learning strategies. Personality and Individual Differences.

[B16-behavsci-16-01368] Hoyle R. H., Gallagher P., Mikulincer M., Shaver P. R., Cooper M. L., Larsen R. J. (2015). The interplay of [ersonality and self-regulation. APA handbook of personality and social psychology: Personality processes and individual differences.

[B17-behavsci-16-01368] Kim K. R., Seo E. H. (2015). The relationship between procrastination and academic performance: A meta-analysis. Personality and Individual Differences.

[B18-behavsci-16-01368] Klingsieck K. B., Grund A., Schmid S., Fries S. (2013). Why students procrastinate: A qualitative approach. Journal of College Student Development.

[B19-behavsci-16-01368] Lay C. H. (1986). At last, my research article on procrastination. Journal of Research in Personality.

[B20-behavsci-16-01368] Lee D. G., Kelly K. R., Edwards J. K. (2006). A closer look at the relationships among trait procrastination, neuroticism, and conscientiousness. Personality and Individual Differences.

[B21-behavsci-16-01368] Liu C., Zhang J. (2025). The effects of aerobic exercise on executive function: A comparative study among active, passive, and non-procrastinating college students. Behavioral Sciences.

[B22-behavsci-16-01368] Logan G. D., Cowan W. B., Davis K. A. (1984). On the ability to inhibit simple and choice reaction time responses. Journal of Experimental Psychology: Human Perception and Performance.

[B23-behavsci-16-01368] Miyake A., Friedman N. P., Emerson M. J., Witzki A. H., Howerter A., Wager T. D. (2000). The unity and diversity of executive functions and their contributions to complex frontal lobe tasks: A latent variable analysis. Cognitive Psychology.

[B24-behavsci-16-01368] Mueller S. T., Piper B. J. (2014). The Psychology Experiment Building Language (PEBL) and PEBL test battery. Journal of Neuroscience Methods.

[B25-behavsci-16-01368] Panadero E. (2017). A review of self-regulated learning: Six models and four directions for research. Frontiers in Psychology.

[B26-behavsci-16-01368] Park S. W., Sperling R. A. (2012). Academic procrastinators and their self-regulation. Psychology.

[B27-behavsci-16-01368] Rabin L. A., Fogel J., Nutter-Upham K. E. (2011). Academic procrastination in college students: The role of self-reported executive function. Journal of Clinical and Experimental Neuropsychology.

[B28-behavsci-16-01368] Rebetez M. M. L., Rochat L., Barsics C., Van der Linden M. (2016). Procrastination as a self-regulation failure: The role of inhibition, negative affect, and gender. Personality & Individual Differences.

[B29-behavsci-16-01368] Richardson M., Abraham C., Bond R. (2012). Psychological correlates of university students’ academic performance: A systematic review and meta-analysis. Psychological Bulletin.

[B30-behavsci-16-01368] Senécal C., Koestner R., Vallerand R. J. (1995). Self-regulation and academic procrastination. The Journal of Social Psychology.

[B31-behavsci-16-01368] Sirois F. M., Melia-Gordon M. L., Pychyl T. A. (2003). “I’ll look after my health, later”: An investigation of procrastination and health. Personality and Individual Differences.

[B32-behavsci-16-01368] Sirois F. M., Pychyl T. A., Sirois F. M., Pychyl T. A. (2016). Procrastination, emotion regulation, and well-being. Procrastination, health, and well-being.

[B33-behavsci-16-01368] Steel P. (2007). The nature of procrastination: A meta-analytic and theoretical review of quintessential self-regulatory failure. Psychological Bulletin.

[B34-behavsci-16-01368] Steel P., Klingsieck K. B. (2016). Academic procrastination: Psychological antecedents revisited. Australian Psychologist.

[B35-behavsci-16-01368] Svartdal F., Dahl T. I., Gamst-Klaussen T., Koppenborg M., Klingsieck K. B. (2020). How study environments foster academic procrastination: Overview and recommendations. Frontiers in Psychology.

[B36-behavsci-16-01368] Svartdal F., Steel P. (2017). Irrational delay revisited: Examining five procrastination scales in a global sample. Frontiers in Psychology.

[B37-behavsci-16-01368] Tice D. M., Bratslavsky E., Baumeister R. F. (2001). Emotional distress regulation takes precedence over impulse control. Journal of Personality and Social Psychology.

[B38-behavsci-16-01368] Toplak M. E., West R. F., Stanovich K. E. (2013). Practitioner review: Do performance-based measures and ratings of executive function assess the same construct?. Journal of Child Psychology and Psychiatry.

[B39-behavsci-16-01368] Verbruggen F., Aron A. R., Band G. P. H., Beste C., Bissett P. G., Brockett A. T., Brown J. W., Chamberlain S. R., Chambers C. D., Colonius H., Colzato L. S., Corneil B. D., Coxon J. P., Dupuis A., Eagle D. M., Garavan H., Greenhouse I., Heathcote A., Huster R. J., Boehler C. N. (2019). A consensus guide to capturing the ability to inhibit actions and impulsive behaviors in the stop-signal task. eLife.

[B40-behavsci-16-01368] Wang Y., Dai M., Wang Z., Jing J. (2025). Investigating the link between trait procrastination and college students’ sleep quality: The mediating role of self-efficacy and executive function. Frontiers in Psychiatry.

[B41-behavsci-16-01368] Watson D. C. (2001). Procrastination and the five-factor model: A facet-level analysis. Personality and Individual Differences.

[B42-behavsci-16-01368] Wieland L. M., Ebner-Priemer U. W., Limberger M. F., Nett U. E. (2021). Predicting delay in goal-directed action: An experience sampling approach uncovering within-person determinants involved in the onset of academic procrastination behavior. Frontiers in Psychology.

[B43-behavsci-16-01368] Zelazo P. D., Carlson S. M. (2012). Hot and cool executive function in childhood and adolescence. Child Development Perspectives.

